# Everybody's Page

**Published:** 1889-12-21

**Authors:** 


### Everybody's Page.

Some ingenious person in Switzerland has invented a watch for the use of the blind. Each figure has a little peg in its centre, which drops when the hour hand reaches the figure. When the owner feels that the peg is down he counts back to twelve, and thus he is enabled to tell the time.

Germany's trade in chemicals seems to be not only very large, but to be rapidly increasing. No money and no energy are spared in the endeavour to make and utilise new discoveries, and the profitable result of the labours of Germans in the field of chemistry are to be seen in the value of the exports of manufactured chemicals.

A tribute of praise is due to the ladies who recently started a first-class magazine in the Braille type for the blind. The periodical is to contain a serial story, short articles, and general information, and, in addition, the publishers have obtained permission from well-known authors to print in Braille selections from their works. Printing in Braille type entails much labour and expense, and it is to be hoped that ample subscriptions will enable these persevering women to carry on their interesting work, which must be such an inestimable boon to the educated blind.

Someone, who evidently felt the truth of what he was writing, has said that a valuable essay might be written on the healing or mitigating power of books on the mind and heart. Most of us at some time of our lives want a good Samaritan to take pity on us, and a good many of us reject the proffered oil and wine for the reason that we do not care to lay bare our wounds which want healing. But unfailing friends do exist whose ministrations any one of us can accept, and these are books; they never betray our secrets, they sympathise, fortify, or rebuke us, and we can turn from one to the other seeking whichever seems to heal our present trouble best. They ask no gratitude, and, above all, they lift us out of our narrow sphere of petty trouble into a larger one of thought and reasoning.

The series of circulars just issued by the Local Government Board, pointing out to the local authorities the extent and the nature of powers which the Legislature has conferred upon them for the purpose of improving the dwellings of the poor, show clearly how easily the most stringent legislation may become a dead letter. The abstract which the circulars give of the powers at the disposal of the authorities is somewhat instructive reading: "The local authorities have been empowered to take the necessary proceedings to prevent any premises in their district unfit for human habitation from being inhabited; to prevent any house in their district from being overcrowded in such a manner as to be prejudicial to the health of the inmates; and to secure the demolition or improvement of buildings which are either themselves unfit for human habitation, or which, by reason of their situation, are the cause of other buildings being rendered insanitary." The necessity which has caused the Local Government Board to take such action clearly demonstrates how signally the permissive powers given by the Act have failed to accomplish their purpose.

If the steps taken by Mr. Ritchie arouse the local authorities to a proper sense of their duty compulsory legislation will be unnecessary. But one difficult problem has yet to be solved in a satisfactory manner, and that is, what is to be done with the houseless tenants whilst the Augæan stable is being cleansed. If the Act is enforced on a large and universal scale, the difficulty of solution will be proportionately increased.

The young grubs of the moths which make such havoc with furs and woollen goods do not when first hatched from the egg, which is generally deposited close to a supply of food, begin to feed on cloths. Their earliest form of food consists of the deserted cases of their ancestors made out of the material on which they fed.

Thousands of people are now flitting or contemplating a flight for a few days for a change. Those who live in large towns, and have not yet decided upon their haven of rest, will do well to avoid going out of the frying-pan into the fire. Mere change of scene is not all that is necessary to the wearied and overtaxed brain; change of mental surroundings and absolute quiet will do more to restore the nerves than any mere change of scene. It is a change to go from London to Brighton, or any other big town, but not necessarily such a change as to do any good. It is astonishing how few people recognise the need of getting the full benefit of a holiday. The theory of our annual flittings has been said to be a desire to seek rest from noise as well as rest from labour.

The rumour, which was spread two years ago, that the berry of a shrub, wrongly called mussaenda, could be employed with such advantage as a substitute for coffee and chicory as to affect seriously the trade in these two latter articles, is now contradicted by the British Consul at Réunion. He informs the public, in his recent report, that the plant alluded to is called "gaertnera," and that the berries do not grow all along the branches, but only in bunches at their ends. The shrub has very few leaves, and grows to about 10ft. in height. So far from endangering the coffee trade, the inferiority in fragrance and colour of the berry will prevent any serious rivalry, especially when coupled with the fact that the shrub only grows in the mountain forests in a wild state, and the picking of the fruit is a troublesome and expensive operation.

Cocoa-nut butter is a new article of domestic consumption, which owes its introduction into use to the discovery by German chemists of a fatty substitute for butter in the cocoa-nut. The United States Consul at Mannheim remarks in his report that the manufactory for this article ere, which has been established for a year, cannot meet the demands made on it. The same authority states that this cocoa-nut butter is not only used in hospitals and other State institutions, but is finding its way into the houses of the poorer and working classes who are unable to buy butter, and prefer the new substance to the oleo-margarines they have hitherto been in the habit of eating. It is said to be free from acids, and easy of digestion, and the taste and smell are not disagreeable. It is of a clear, whitish colour, and better adapted for cooking than for table purposes. This latter statement we can readily believe.

Every dog has his day, and according to Dr. M. A. Starr the phrenologists have had theirs. We confess a lingering affection for the sage-looking little heads marked out in patterns which we see in the windows of these wise men, and recall the modest fee in return for which we received so much information which was amusing, if not valuable. Dr. Starr, in his article in the *Popular Science Monthly*, says that it is impossible to draw any conclusion from the size or shape of the head as to the extent or surface of the brain, and so as to the mental capacity. It is absurd to judge of the brain surface by either the size of the head or the extent of the superficial irregular surface which is covered by the skull, without taking into consideration the number of folds and the depth of the creases. "For a little brain with many deep folds may really, when spread out, have a larger surface than a large brain with few shallow folds."

